# Murine GPRC6A Mediates Cellular Responses to L-Amino Acids, but Not Osteocalcin Variants

**DOI:** 10.1371/journal.pone.0146846

**Published:** 2016-01-19

**Authors:** Patricia Rueda, Elizabeth Harley, Yao Lu, Gregory D. Stewart, Stewart Fabb, Natalie Diepenhorst, Béatrice Cremers, Marie-Hélène Rouillon, Isabelle Wehrle, Anne Geant, Gwladys Lamarche, Katie Leach, William N. Charman, Arthur Christopoulos, Roger J. Summers, Patrick M. Sexton, Christopher J. Langmead

**Affiliations:** 1 Drug Discovery Biology, Monash Institute of Pharmaceutical Sciences, Monash University, Parkville, Victoria 3052, Australia; 2 Institut de Recherches Servier, Suresnes, France; Omaha Veterans Affairs Medical Center, UNITED STATES

## Abstract

Phenotyping of *Gprc6a* KO mice has shown that this promiscuous class C G protein coupled receptor is variously involved in regulation of metabolism, inflammation and endocrine function. Such effects are described as mediated by extracellular calcium, L-amino acids, the bone-derived peptide osteocalcin (OCN) and the male hormone testosterone, introducing the concept of a bone-energy-metabolism-reproduction functional crosstalk mediated by GPRC6A. However, whilst the calcium and L-amino acid-sensing properties of GPRC6A are well established, verification of activity of osteocalcin at both human and mouse GPRC6A *in vitro* has proven somewhat elusive. This study characterises the *in vitro* pharmacology of mouse GPRC6A in response to its putative ligands in both recombinant and endogenous GPRC6A-expressing cells. Using cell signalling, and glucagon-like peptide (GLP)-1 and insulin release assays, our results confirm that basic L-amino acids act as agonists of the murine GPRC6A receptor in both recombinant cells and immortalised entero-endocrine and pancreatic β-cells. In contrast, our studies do not support a role for OCN as a direct ligand for mouse GPRC6A, suggesting that the reported *in vivo* effects of OCN that require GPRC6A may be indirect, rather than *via* direct activation of the receptor.

## Introduction

GPRC6A is a class C G protein-coupled receptor (GPCR) that has been cloned from human, mouse and rat. The receptor was initially deorphanised by fusion of its large N-terminal domain to the heptahelical and C-terminal region of the related goldfish 5.24 receptor [1]. This construct, and full length GPRC6A, mediates responses to L-amino acids, notably basic amino acids such as arginine, ornithine and lysine. These ligands are thought to bind in the N-terminal domain of the receptor [2] in a manner analogous to the binding of L-glutamate to metabotropic glutamate receptors. Small molecules, such as NPS-2143, calindol and indole-based ligands, have been shown to bind in the 7TM domain of the receptor to act as negative allosteric modulators of GPRC6A [3, 4].

Recombinant GPRC6A expression studies have proven less than straightforward since the human receptor expresses poorly at the cell surface, due to an intracellular retention motif in the third intracellular loop [5]. Cell signalling studies with the murine orthologue suggest that the receptor couples primarily *via* the Gα_q/11_ pathway to increase inositol phosphate production and mobilise intracellular calcium [1, 6]; however, the efficiency of coupling is often poor but can be substantially improved by co-expression of exogenous or mutated G proteins e.g. Gα_qG66D_ [7]. Other studies have indicated that GPRC6A may increase cyclic AMP and/or phosphorylation of extracellular signal-regulated kinase-1/2 (ERK1/2) [8–12]. Thus the preferred downstream signal transduction pathway(s) of GPRC6A are not well defined and may be dependent on species and cell background [7, 13].

The pharmacology of GPRC6A is of interest due to recent studies that have implicated the receptor in several metabolic, endocrine and inflammatory processes [11, 12, 14–19]. There has been some debate as to the extent to which GPRC6A regulates metabolic function; one *Gprc6a* KO mouse strain displays manifestations of metabolic syndrome, increased serum glucose levels after an overnight fast as well as impaired insulin sensitivity [16]. However, a different KO mouse displayed a subtler phenotype, with no evidence of impaired glucose handling or insulin sensitivity (and disruptions in glucose metabolism only when the mice were fed a high fat diet, a phenotype that could also result directly from obesity, rather than the absence of GPRC6A itself) [18]. As a result, there remain questions as to the role of GPRC6A in controlling metabolic and endocrine functions.

Additional interest in GPRC6A has emerged due to several studies reporting that it mediates the metabolic and endocrine effects of the de-carboxylated form of the osteoblast-derived peptide, osteocalcin (OCN). This peptide has been shown to control energy expenditure regulated by the pancreas [20]; mice lacking OCN display decreased pancreatic cell proliferation, glucose intolerance and insulin resistance. OCN, when injected into the peritoneal cavity of mice, increases circulating insulin levels in WT but not in *Gprc6a* KO mice [12]. OCN has also been shown to increase pancreatic β-cell proliferation, insulin release from pancreatic islets, insulin sensitivity and energy expenditure and testosterone synthesis in the gonads in mice [20, 21]. Furthermore, OCN-mediated insulin and glucagon-like peptide (GLP)-1 release have also been reported in immortalised cell lines (e.g. pancreatic β-cells) and have been blocked by siRNA targeting *Gprc6a* [12, 22]. These data, together with the lack of OCN efficacy in *Gprc6a* KO mice, both *in vivo* and *ex vivo*, has led to suggestions that de-carboxylated OCN acts directly via GPRC6A to exert its metabolic and endocrine effects. Despite this evidence, a direct action of OCN on the mouse receptor has been hard to confirm. Several studies have demonstrated that de-carboxylated OCN can stimulate cAMP accumulation or ERK1/2 phosphorylation in HEK293 cells recombinantly expressing GPRC6A (though the species tested is not always stated [8, 10–12]), suggesting that the signalling of the receptor may not be limited to Gα_q_ linked pathways. However, another study in cells recombinantly expressing mouse GPRC6A failed to replicate either the agonist activity of OCN, or the effects on cAMP signalling [13], leading the authors to conclude that GPRC6A is primarily a Gα_q_-coupled, basic L-amino acid receptor.

The aim of this study was to determine whether de-carboxylated OCN is an agonist of GPRC6A in either recombinant or endogenously expressing cells, using a range of cell signalling and phenotypic assays. Lack of expression precluded study of the human receptor, but our results in HEK293 cells suggest that murine GPRC6A is an L-amino acid receptor that preferentially increases inositol phosphates and stimulates intracellular calcium mobilisation. No cAMP signalling or ERK1/2 phosphorylation was evident, and no agonistic or modulatory effects of de-carboxylated OCN in any GPRC6A signalling assay were detected. We also confirmed an effect of L-ornithine to promote GLP-1 release and glucose-sensitive insulin secretion in GPRC6A-expressing immortalised entero-endocrine and pancreatic β-cells, respectively, but did not observe any effect of OCN variants. Only in rat insulinoma INS-1(832) cells that did not express *Gprc6a* transcript were we able to observe any cellular response to OCN variants, specifically a change in cellular impedance. Taken together, our results suggest that the reported mouse GPRC6A-dependent effects of OCN may result from indirect, rather than direct activation of the receptor.

## Materials and Methods

### Materials

RPMI 1640 (no glucose); Dulbecco’s Modified Eagle Medium (DMEM); DMEM (high glucose, no glutamine, lysine, or arginine); sodium pyruvate; penicillin/streptomycin; foetal bovine serum and lipofectamine LTX (GIBCO-Invitrogen-Life Technologies, Carlsbad, CA). β-Mercaptoethanol, glucose, blasticidin S, ATP, forskolin, IBMX and L-amino acids (Sigma Aldrich, St. Louis, MO); hygromycin B (Roche, Basel, Switzerland); tetracycline (Fluka, St. Gallen, Switzerland); puromycin (InvivoGen (San Diego, CA); anti-cmyc 9E10 antibody (Santa Cruz, Dallas, Texas) and goat-anti mouse-PE antibody (GIBCO-Invitrogen-Life technologies, Carlsbad, CA); SYTOX Red and Fluo-4 AM (Molecular Probes, Eugene, Oregon). GLUTag cells were kindly provided by Dr. Daniel J. Drucker (Lunenfeld-Tanenbaum Research Institute, Mount Sinai Hospital, Toronto, ON, Canada), MIN6 cells by Dr. Jun-ichi Miyazaki (Osaka University, Osaka, Japan) and INS-1 (832) cells by Dr. Christopher Newgard (Sarah W. Stedman Nutrition and Metabolism Center, Duke University, Durham, NC).

### Osteocalcin (OCN) variants

Purified bovine OCN was purchased from Meridian Life Science (Memphis, TN, USA). Human and mouse synthetic OCN (C-terminal acid and amide) were purchased from Federation Bioscience (Melbourne, VIC, Australia). Briefly, peptides were synthesised using Fmoc-solid phase, purified, folded and the single disulphide bond formed by aeration. Although the 3D structure of the peptides obtained was not determined, the observed molecular weight was consistent with the formation of the disulphide bond. Synthetic peptides were >98% pure as assessed by reverse phase-HPLC and MALDI-TOF mass spectrometry. Mouse OCN was expressed recombinantly as an N-terminal GST-tag fusion protein as previously described [23]. Briefly, the fusion protein was expressed in BL21 (DE3) Codon Plus *E*. *coli* (Stratagene) in 2YT media after induction with 1 mM IPTG, 37°C for 3h. Cultures were harvested and pellets were lysed using B-PER (Pierce). The soluble lysate fraction was collected after centrifugation at 10000 g at 4°C for 15 min and purified using GST spin purification kits (Peirce). The fusion protein was eluted using 3 mg/ml reduced glutathione, 125 mM Tris, 150 mM NaCl (pH 8.0) and subsequently cleaved with thrombin (Novagen) according to the manufacturers protocol overnight at 4°C with gentle rocking. The cleaved protein of interest was isolated using reverse phase-HPLC (Lumina C8 (Phenoenex) column and a Prep LC system (Waters)). Mouse OCN was eluted at 24 min (60% acetonitrile), confirmed by ESI-TOF mass spectrometry (Agilent Technologies) and yields of 2mg/L culture were routinely achieved.

### Cell line generation

Mouse and human GPRC6A (mGPRC6A and hGPRC6A) DNA sequences were obtained from Genecopoeia in pDONR vectors. These clones, both with and without an N-terminal c-*myc* tag preceded by the mGlu5 receptor signal peptide [13] (to detect receptor expression by immunofluorescence), were assembled using PCR and transferred into the pCDNA 5/FRT/TO vector (Invitrogen) or pEF-IRES-puro6 vector [24] (kindly provided by Sarawut Jitrapakdee, Department of Biochemistry, University of Adelaide, Adelaide SA 5005). Gα_q_ sequence in pcDNA3.1+ was purchased from the cDNA Resource centre (Bloomsburg, Pennsylvania, USA) and substitution of Gly 66 for Asp was made using Quickchange II site-directed mutagenesis (Agilent Technologies, USA) with the following primers; sense 5’-atgagaatcatccatgggtcagattactctgatgaagataaaaggg-3’, antisense 5’- cccttttatcttcatcagagtaat ctgacccatggatgattctcat-3’.

pcDNA 5/FRT/TO constructs were isogenically integrated into a FlpIn-TREx-HEK293 cell line (Life Technologies). FlpIn-TREx-HEK293-mGPRC6A and FlpIn-TREx-HEK293-hGPRC6A cells were then selected using 600 μg/ml hygromycin and 5 μg/ml blasticidin and grown in culture medium consisting of Dulbecco’s Modified Eagle Medium (DMEM), supplemented with 10% (v/v) foetal bovine serum (FBS) at 37°C in 5% CO_2_ and were sub-cultured in a 1:3 ratio every 3–4 days. Cells were cultured overnight in the presence of 1 μg/ml tetracycline to induce GPRC6A expression. An additional, higher expressing mGPRC6A cell line was generated for GPRC6A assays in which L-amino acids did not show activity (vide infra); pIRES-puro6 constructs were transfected into FlpIn-TREx-HEK293 cells using Lipofectamine LTX following the manufacturer’s guidelines. 48 h after transfection, cells were selected using 3 μg/ml puromycin and grown as above.

### Flow cytometry

Cells were harvested with PBS-EDTA and 10^5^ cells / well were seeded into a V-bottom 96-well plate. Cells were incubated in blocking buffer (PBS with 0.01% azide, 5% goat serum, 2 mM EDTA) for 30 min at 4°C. Primary antibody (anti-c-*myc*, 9E10) was added at a 1 μg/ml final concentration and cells were incubated for 1 h at 4°C. After washing cells twice with wash buffer (PBS, 0.01% sodium azide, 0.5% goat serum (v/v), 2 mM EDTA), cells were incubated with 1 μg/ml of secondary antibody (goat anti-mouse-PE) diluted in wash buffer plus SYTOX Red dead cell stain (1:1000; v/v) for 45 min at 4°C. Cells were then washed twice with wash buffer and resuspended in PBS into BD Falcon tube with cell strainer cap. Cells were then analysed for PE and SYTOX Red fluorescence using a BD FACSCanto II flow cytometer. For analysis, the population of interest was gated on a FSC/SSC density plot. Live cells were selected and analysed for PE stain intensity and/or percentage over untransfected control cells.

### Quantitative PCR

Cells (cultured to 75% confluency and washed with PBS) or islets were treated with lysis buffer from RNAeasy plus kit (Qiagen). RNA was extracted according to the manufacturer’s protocol. Samples were eluted in 30 μl of ddH_2_O and RNA concentration determined using the Nanodrop 2000 (Thermo Scientific). cDNA was synthesised from 1 μg total RNA using the M-MLV RT polymerase (or water for the RT- controls) and random hexamers by incubating the samples at 60°C for 5 min followed by 1 h at 42°C and 15 minutes at 90°C. cDNA samples were diluted 1 in 5 and 1 μl of each sample was used to perform real time PCR using Sybr Green or a specific Taqman assay for mouse *Gprc6a* from Life technologies (Mm01192898_m1) using mouse actin as a reference gene. Three known dilutions of plasmid containing the mouse or rat *Gprc6a* sequence were run in parallel to determine the primer or Taqman assay efficiency. For Sybr green assays, the primer pairs used were: mouseHprt: mHprt_f 5’ctggtgaaaaggacctctcg3’, mHprt_r 5’tgaagtactcattatagtcaagggca3’; mouseGprc6a: mGprc6a_f 5’tcatgccacaggtgagttatgaat3’, mGprc6a_r 5’ggcaccaatccagttccatc3’; ratHprt: rHprt_f 5’ctggtgaaaaggacctctcg3’, rHprt_r 5’tgaagtgctcattatagtcaagggca3’; ratGprc6a: rGprc6a_f 5’tcatgccacaggtgagttatgaat3’, rGprc6a_r 5’ggcaccaacccagttccat3’. Results were expressed as ΔCt of the gene of interest versus the reference gene for every sample.

### Ca^2+^_i_ mobilisation assays

FlpIn-TREx-HEK293-mGPRC6A cells were seeded into T75 flasks at 70% confluency. Cells were transfected with 15μg of plasmid DNA (Gα_q_ or Gα_qG66D_) with Lipofectamine LTX according to manufacturer's instructions, then incubated for 24h at 37°C in 5% CO_2_. Cells were plated into 96-well plates (10^5^ cells/well) and equilibrated at 37°C in 5% CO_2_ for 4-6h to adhere, whereupon cells were serum starved overnight at 37°C in 5% CO_2_. For the assay, cells were washed twice in Ca^2+^ assay buffer (150 mM NaCl, 2.6 mM KCl, 1.2 mM MgCl_2_, 10 mM dextrose, 10 mM HEPES, 2.2 mM CaCl_2_, 0.5% (w/v) BSA and 4 mM probenecid; pH 7.4). Buffer was then replaced with Ca^2+^ assay buffer containing 1μM Fluo-4-AM and incubated for 1h at 37°C in 5% CO_2_. Cells were washed twice more and replaced with Ca^2+^ assay buffer at 37°C. Serial dilutions of test agonists were added by injection in a Flexstation^™^ (Molecular Devices) and fluorescence was measured at 485nm excitation and 520nm emission wavelengths. Agonist responses were normalised to the peak of response to 100 μM ATP.

### Inositol phosphate accumulation assays

Inositol phosphate (IP_X_) accumulation experiments were performed using the IP-One HTRF^®^ assay kit (Cisbio, France). Briefly, 24h after transfection with G proteins (as above), 10^5^ cells were seeded per well in PDL-coated, 96-well white plates and incubated overnight. Cells were then washed 2 x 2h at 37°C in HBSS buffer (pH 7.2) containing 20 mM HEPES, 3.5 mM NaHCO_3_, 1 mM CaCl_2_ and 0.1% (w/v) BSA. Test compounds were then prepared in the kit stimulation buffer containing 2 mM CaCl_2_; cells were then incubated with 70 μl of drugs for 30 min, after which 15 μl of both IP1-D2 and Ab-Cryp were added to the cells, followed by a 1 h incubation at RT. Plates were read using an Envision plate reader (Perkin Elmer), using an HTRF protocol. Results were calculated from the 665nm / 620nm HTRF ratio (Ratio = A_665nm_/A_620nm_ x 10^4^) and expressed as a percentage of the maximal response obtained.

### Extracellular signal-regulated kinase (ERK) 1/2 phosphorylation assays

ERK1/2 phosphorylation time course experiments were performed in FlpIn-TREx-HEK293-mGPRC6A cells transfected with G proteins (as above) using the SureFire Alphascreen kit. Cells were plated in 96-well plates; after 6h incubation at 37°C in 5% CO_2_, cells were washed twice with PBS and incubated in serum- and arginine / lysine-free DMEM at 37°C overnight to reduce basal ERK1/2 phosphorylation. Cells were then stimulated with ligand at various time points and incubated at 37°C in 5% CO_2_. 10% FBS (v/v) was used as a positive control. The reaction was terminated by removal of compounds and lysis of cells with SureFire lysis buffer (TGR Biosciences). The lysates were then treated as per the SureFire ERK1/2 kit instructions. Plates were incubated in the dark at 37°C for 1 h, then left to equilibrate with the ambient temperature before the fluorescence signal was measured using a Fusion plate reader (PerkinElmer) with standard AlphaScreen settings. Data were normalised to the maximal response elicited by 10% FBS (v/v) at the same time point.

### Cyclic AMP accumulation assays

HEK293-mGPRC6A cells were seeded at a density of 10^5^ cells per well into PDL-coated 96-well plates and incubated overnight in DMEM, supplemented with 10% FBS (v/v) at 37°C in 5% CO_2_. Cells were then incubated in 90 μl of stimulation buffer (HBSS containing 5 mM HEPES, 0.1% BSA (w/v), 0.5 mM IBMX; pH 7.4) for 30 min at 37°C. Test compounds (10 μl at 10 x final) were added and cells were incubated for an additional 30 min at 37°C. The medium was removed and cells were incubated in 50 μl of ice-cold 100% ethanol. Once evaporated, 100 μl of lysis buffer (5 mM HEPES, 0.1% BSA (w/v), 0.3% Tween-20; pH 7.4) was added to the cells and samples were stored at -20°C until further analysis. Samples were analysed using the Alphascreen^®^ cAMP assay kit (Perkin Elmer) following manufacturer instructions. Briefly, 10 μl of lysates were added to a 384-well Optiplate. After addition of 5 μl of acceptor bead mix, plate was incubated at RT for 30 min followed by addition of 15 μl of donor bead mix. After overnight (16 h) incubation at RT, plates were read using the Envision plate reader.

### Label-free cell impedance assays

2.5 x 10^7^ FlpIn-TREx-HEK293-mGPRC6A cells were seeded in a T175 flask and incubated overnight at 37°C in 5% CO_2_. Cells were then transfected with 35 μg of plasmid DNA (Gα_qG66D_) with Lipofectamine LTX according to manufacturer's instructions, then incubated for 24h at 37°C in 5% CO_2_. Prior to the experiment, a background reading for each E-plate 96 (Bio-Rad, CA, USA) was measured after addition of 100 μl DMEM + 10% FBS per well. Cells were recovered using Versene and seeded at a 10^5^ cells/well density in 200 μl DMEM + 10% FBS (containing 1 μg/ml tetracycline where indicated). Cells were washed 2 x 2 h in HBSS buffer (pH 7.2) containing 20 mM HEPES, 1 mM CaCl_2_ and 0.1% BSA (w/v) in a total volume of 180 μl. Test compounds (20 μl at 10x final concentration) were added to the plate and cell impedance was monitored for 3 h. Cell index was normalised to the buffer response (minus the baseline response at zero time point).

For INS-1 cells, 100 μl of medium (RPMI 1640 no glucose, 10% FBS, 55 μM β-mercaptoethanol, 1 mM sodium pyruvate, 10 mM HEPES, 100 U/ml penicillin, 100 μg/ml streptomycin, 11.1 mM glucose) was added per well to an E-plate 96 and a background reading taken. Cells were seeded at 2 x 10^4^ / well and the plates incubated in the instrument for 48 h. On the day of the experiment, media was changed to EBSS buffer (180 μl; 2.8 mM glucose, 1.8 mM CaCl_2_, 5.3 mM KCl, 0.8 mM MgSO_4_, 117 mM NaCl, 26 mM NaHCO_3_, 1 mM NaH_2_PO_4_). After 2 h incubation, cells were stimulated with 20 μl test compound (10 x final) prepared in the same buffer. Data capture and analysis were as for HEK293 cells.

### GLP-1 release assays

Intestinal L-cell model GLUTag cells were seeded in 24-well plates coated with Matrigel and incubated for 6 days in DMEM with 10% FBS, 100 U/ml penicillin, 100 μg/ml streptomycin at 37°C in 5% CO_2_ and grown for 4–5 days. Where indicated, cells were washed (1 or 2 x 1h) with Krebs-HEPES buffer (20 mM HEPES, 118 mM NaCl, 4.7 mM KCl, 1.2 mM MgSO_4_, 5 mM NaHCO_3_, 1.2 mM KH_2_PO_4_, 2.5 mM CaCl_2_, 5.5 mM glucose, 0.2% BSA (w/v), pH 7.2) containing vildagliptin (300 μM) prior to addition of test compounds in the same buffer. After 2 h incubation at 37°C in 5% CO_2_, the supernatant was recovered and centrifuged at 500 rpm for 5 min. Where used, NPS-2143 pre-treatment was for 30 min.

GLP-1 content in the cell supernatant was measured by ELISA (EGLP-35K, Millipore), according to manufacturer’s instructions after overnight incubation of 15 μl supernatant in 100 μl Krebs-HEPES buffer at 4°C. After 5 x washes, 200 μl of detection conjugate was added and incubated for 2 h at RT. After 3 x washes the reaction was developed with 200 μl of diluted substrate (1:200) for 20 min and terminated by addition of 50 μl stop solution. After 5 min, fluorescence was then measured using 355 nm excitation and 460 nm emission, transforming the obtained signal into pmol GLP-1/well/ml and corrected for GLUTag cell protein content as determined using the method of Bradford (1976).

### Insulin release assays

MIN6 or βTC-6 cells were seeded in 24-well plates at 170,000 cells per well in DMEM (containing 4.5g/L glucose, 10 mM HEPES, 100 U/ml penicillin, 100 μg/ml streptomycin and 15% de-complemented FBS) and incubated at 37°C / 5% CO_2_ for 72 h. Cells were then washed with 500 μl PBS and incubated in 500 μl deprivation media (DMEM with zero glucose, 10 mM HEPES, 1 mM Na-pyruvate, 100 U/ml penicillin, 100 μg/ml streptomycin, 15% de-complemented FBS) for 2 h at 37°C. Media was aspirated and after washing with 500 μl PBS, cells were treated with 250 μl of Krebs buffer (250 mM NaCl, 5.9 mM KCl, 1.2 mM MgCl_2_, 1.3 mM CaCl_2_, 25 mM HEPES, 0.1% BSA (w/v), and 2 mM or 16.7 mM glucose; pH 7.4) in the presence or absence of test compound. After incubation for 2 h at 37°C, the assay was terminated by placing the plates on ice and supernatants were transferred to a 96-well plate in preparation for insulin ELISA (Alpco) following manufacturer’s instructions. The cell monolayer was washed twice with 500 μl of PBS and then lysed using 250 μl of 1 N NaOH prior to protein measurement by the method of Bradford (1976).

Islets were collagenase-isolated from mice (10 x C57Bl/6; 12–13 weeks; Charles River) and incubated overnight in RPMI (with 10% FBS (v/v), 10 mM HEPES, 100 U/ml penicillin, 100 μg/ml streptomycin, sodium pyruvate and 10 mM glucose) at 37°C in 5% CO_2_. Islets were seeded into a 96-well plate (4 / well) in Kreb’s buffer containing 2.8 mM glucose and washed twice with the same buffer. 200 μl test drugs were added in Kreb’s buffer with the required concentration of glucose and incubated at 37°C in 5% CO_2_ for 90 min. The plate was centrifuged for 10 s at 200 g, supernatant recovered and insulin levels detected as described above. This protocol was approved by the IDRS (Institut de Recherches Servier) Institutional Animal Care and Use Committee.

### Data analysis

Concentration-response data were analysed using a three or four parameter logistic function (Motulsky & Christopoulos, 2004) to generate estimates of agonist potency (LogEC_50_). Data for all HEK293 assays represent the mean ± SEM of at least three independent experiments (except cell impedance, n = 2). For GLP-1 release, the data represent mean ± SEM of three to five independent experiments (except for OCN, wash; n = 2). For insulin release assays, data represent the mean ± SEM of three independent experiments (except for islet studies; n = 2). Insulin release data were normalised to the response to vehicle in either low or high [glucose]. Statistical analysis for GLP-1 release data were performed by one-way ANOVA, followed by Sidak’s multiple comparisons test (treating wash / no wash datasets separately). Statistical analyses for insulin release data were performed by two-way ANOVA (with [glucose] and drug treatment as group variables), followed by Sidak’s multiple comparisons test. Where only high [glucose] groups were evaluated, statistical analysis was by one-way ANOVA followed by Sidak’s multiple comparisons test.

## Results

### Recombinant expression of GPRC6A orthologues

Using the tetracycline-inducible FlpIn technology, cells expressing both untagged and N-terminally c-*myc*-tagged GPRC6A were generated in HEK293 cells. This resulted in a stable cell line with robust cell-surface expression of mouse but not human GPRC6A, as determined by FACS analysis (S1 Fig). Neither untransfected nor un-induced FlpIn-TREx-HEK293 cells showed GPRC6A expression (S1 Fig and *data not shown*). Cell lines were also generated using a bicistronic pIRES-puro6 expression vector expressing a puromycin resistance gene. Despite higher expression for mouse GPRC6A compared to the FlpIn-TREx-HEK293 cell line, this approach still failed to yield cell surface expression of the human receptor (S1 Fig). During the preparation of this manuscript, a study demonstrated, using chimeric human/mouse GPRC6A constructs, that a missing “RKLP” motif in the third intracellular loop causes the intracellular retention and lack of cell-surface function of the human GPRC6A orthologue [5]. As a result of the failure to express the human receptor at the cell surface, all subsequent functional studies were performed in HEK293 cells stably expressing mouse GPRC6A.

### Recombinant cell signalling

**Fig 1:**
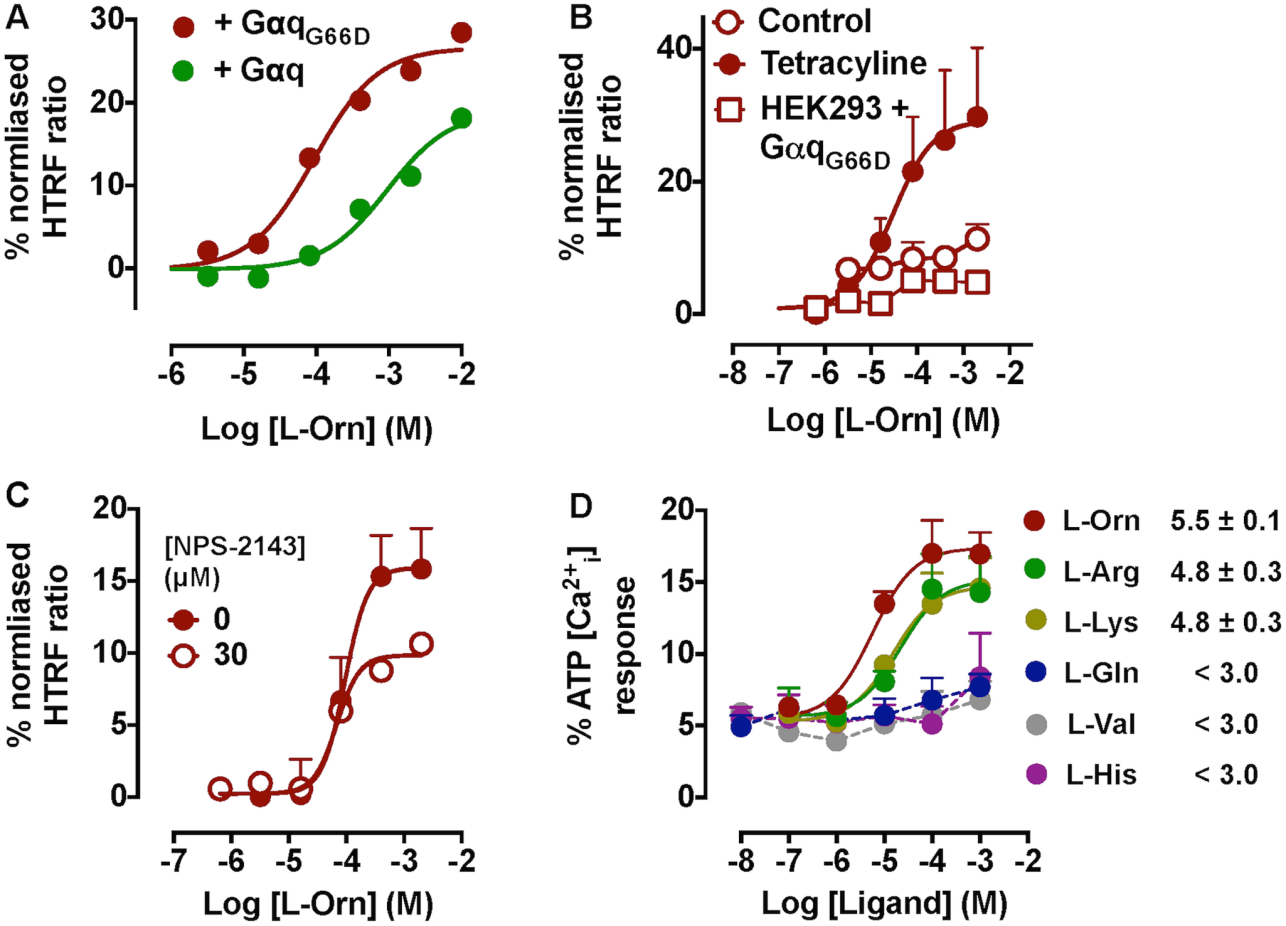
Calcium mobilisation and inositol phosphate accumulation in FlpIn-TREx-HEK293-mGPRC6A cells. (A) Inositol phosphate accumulation responses to L-ornithine in FlpIn-TREx-HEK293-mGPRC6A cells with co-expression of Gα_q_ or Gα_qG66D_. (B) The L-ornithine response is specific to FlpIn-TREx-HEK293 cells (co-expressing Gα_qG66D_) induced to express mGPRC6A with tetracycline, but absent in non-tetracycline induced or parental HEK293 cells. (C) GPRC6A-mediated inositol phosphate accumulation can be inhibited with the antagonist, NPS-2143 (30 μM). (D) Ca^2+^ mobilisation induced by various L-amino acids in FlpIn-TREx-HEK293-mGPRC6A cells co-expressing Gα_qG66D_. The rank order of potency is L-ornithine > L-arginine = L-lysine (values quoted represent pEC_50_ ± SEM). Similar responses were observed with Gα_q_ co-expression (*data not shown*).

Since previous signalling studies suggest that GPRC6A preferentially couples to the Gα_q/11_ pathway, we first assessed HEK293-mGPRC6A cells in assays of calcium mobilisation and inositol phosphate accumulation. In spite of robust receptor expression levels (S1 Fig), only very small responses to L-amino acids were observed (*data not shown*). However, upon transient expression of Gα_q_ proteins (either WT or the promiscuous G66D mutant), robust agonist activity to basic amino acids was detected in both calcium mobilisation and inositol phosphate accumulation assays (Fig 1). Co-expression of Gα_qG66D_ yielded a ten-fold higher potency (pEC_50_ = 4.0 ± 0.2) for L-ornithine in inositol phosphate accumulation assays compared to co-expression of wild-type Gα_q_ (pEC_50_ = 3.0 ± 0.5; Fig 1A), likely reflecting improved stimulus-response coupling. The observed signal was specific to GPRC6A, as G protein-transfected parental HEK293 cells and non-tetracycline-induced FlpIn-TREx-HEK293-mGPRC6A cells did not respond to L-amino acids (Fig 1B). Furthermore, inositol phosphate accumulation in response to L-ornithine was sensitive to application of the GPRC6A antagonist, NPS-2143 (Fig 1C). In calcium mobilisation assays, the rank order of potency was L-ornithine > L-arginine = L-lysine (Fig 1D), in agreement with previous studies [7], with little or no activity detected for L-glutamine, L-valine or L-histidine.

Concomitantly to evaluating amino acid pharmacology of mGPRC6A, we profiled variants of OCN in the assays. OCN contains three γ-carboxyglutamic acid residues, thus existing in different forms based on the degree of carboxylation, which determines its affinity for the extracellular bone matrix [25–27]. The degree of carboxylation could be a mechanism by which the OCN function is controlled, although this theory is still not proven. Although in mice the metabolic effects of OCN were observed exclusively when using the de-carboxylated form, in middle-aged male subjects, levels of both de-carboxylated and carboxylated forms of OCN are inversely associated with plasma glucose level, with the latter more closely related to improved insulin sensitivity rather than increased β-cell function [28].

**Fig 2:**
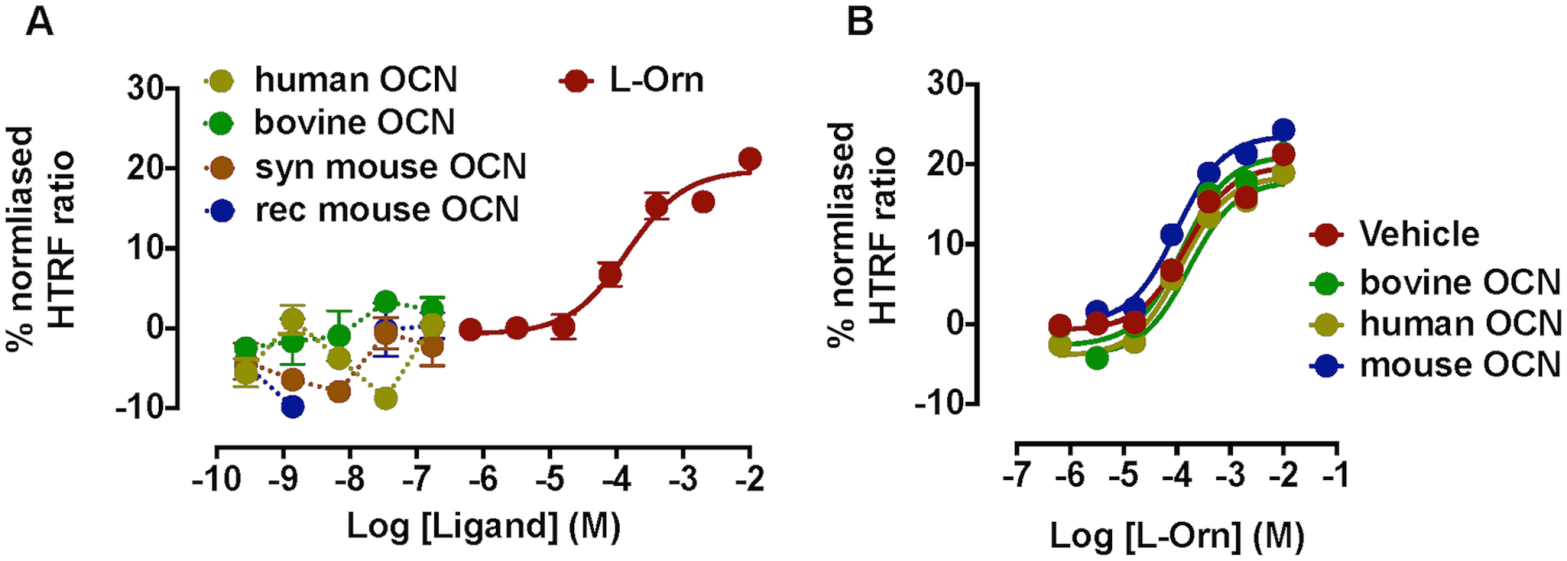
Effect of OCN on inositol phosphate accumulation in FlpIn-TREx-HEK293-mGPRC6A cells. (A) Variants of the putative GPRC6A peptide ligand osteocalcin (OCN) fail to induce inositol phosphate accumulation in FlpIn-TREx-HEK293-mGPRC6A cells co-expressing Gα_qG66D_. (B) OCN variants (40 ng/ml) do not modulate L-ornithine-stimulated inositol phosphate accumulation at mGPRC6A (OCN variants as described in Table 1).

To obviate any species differences we profiled synthetic de-carboxylated human OCN, synthetic and recombinant de-carboxylated mouse OCN (synthetic forms both acidic and amidated) as well as partially carboxylated OCN from bovine bone (Table 1). Multiple OCN variants were tested in the mGPRC6A inositol phosphate accumulation or calcium mobilisation assays (with both transiently transfected Gα_q_ and Gα_qG66D_). As shown in Fig 2A (Gα_qG66D_ transfected cells in inositol phosphate accumulation assays), no response was detected to any OCN variant. Similar results were obtained in cells transiently transfected with Gα_q_ and in calcium mobilisation assays (*data not shown*). We then investigated the possibility of an indirect effect of OCN on mGPRC6A function; however OCN variants (40 ng/ml) did not modulate the potency or maximal response to L-ornithine in assays of inositol phosphate accumulation (Fig 2B).

**Table 1:** Osteocalcin (OCN) variants used in this study.

Species	Method	Source	Carboxylation Status	Sequence
Bovine	Purified from bone	Meridian Life Science	Carboxylated / under-carboxylated (Benton 1995, Price 1976)	N/A
Human	Recombinant	Novus Biological	De-carboxylated	N/A [Cat. No. NP_954642.1; 1 a.a.—100 a.a.; includes ~ 26kDa N-terminal GST tag]
Human	Synthetic	Federation Bioscience	De-carboxylated (amide and acid)	YLYQWLGAPVPYPDPLEPRREVCELNPDCDELADHIGFQEAYRRFYGPV-NH2/-COOH
Mouse	Recombinant	Produced in house	De-carboxylated	GSPEF-YLGASVPSPDPLEPTREQCELNPACDELSDQYGLKTAYKRIYGITI

Although OCN variants did not display activity in assays classically linked to Gα_q_ activation in which L-amino acids displayed robust agonism, previous studies profiling OCN at GPRC6A have also examined both cAMP accumulation and ERK1/2 phosphorylation. Therefore we determined the effects of both L-ornithine and OCN variants in assays for these endpoints in FlpIn-TREx-HEK293-mGPRC6A cells.

**Fig 3:**
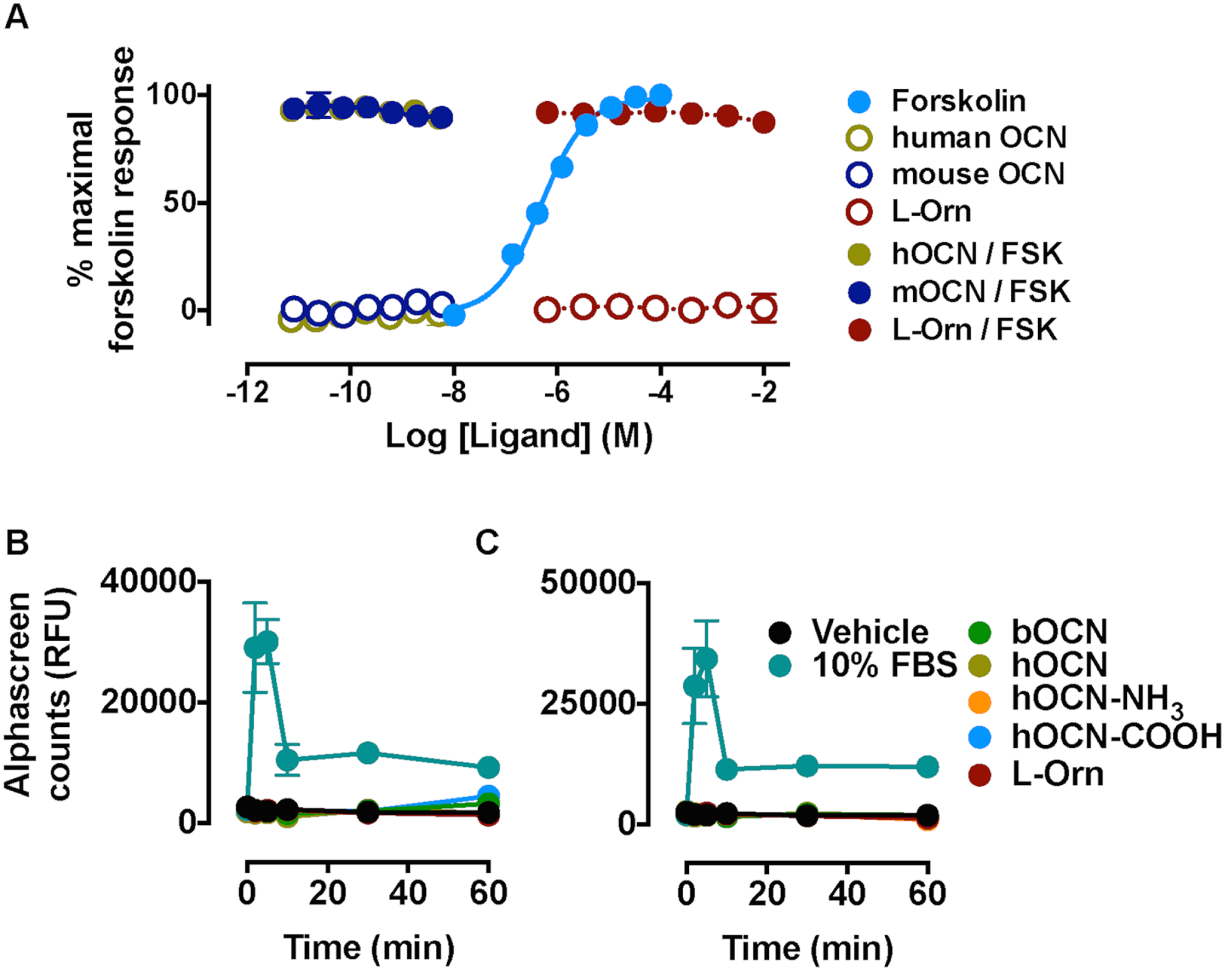
Modulation of cAMP levels and ERK1/2 phosphorylation in FlpIn-TREx-HEK293-mGPRC6A cells. (A) OCN variants do not stimulate cAMP accumulation in FlpIn-TREx-HEK293 cells stably expressing mGPRC6A (open circles). Neither L-ornithine nor OCN variants inhibit forksolin (3 μM)-stimulated cAMP accumulation in the same cell line (filled circles; OCN variants as described in Table 1). (B) Neither L-ornithine (1 mM) nor OCN variants (40 ng/ml) stimulate ERK1/2 phosphorylation in FlpIn-TREx-HEK293-mGPRC6A cells; (C) a similar lack of activity was observed in cells co-expressing Gα_qG66D_.

Despite robust increases in cAMP levels with forskolin in FlpIn-TREx-HEK293-mGPRC6A cells, none of the putative GPRC6A agonists, including OCN variants, increased cAMP levels in their own right. Furthermore, neither L-ornithine nor OCN variants inhibited cAMP levels stimulated by forskolin (3 μM; Fig 3A). Similar results were observed in the higher-expressing pIRES-puro6 cell line (S2 Fig). Furthermore, although the positive control (10% FBS) produced a robust increase in ERK1/2 phosphorylation in FlpIn-TREx-HEK293-mGPRC6A cells, none of the putative GPRC6A agonists had any effect on ERK1/2 phosphorylation (in the absence or presence of transiently expressed Gα_qG66D_; Fig 3B and 3C, respectively).

**Fig 4:**
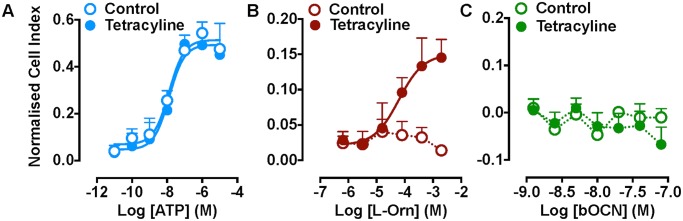
Measurement of cellular impedance in FlpIn-TREx-HEK293-mGPRC6A cells. (A) ATP (acting via endogenous P2Y receptors) increases cellular impedance in both tetracycline-induced and un-induced FlpIn-TREx-HEK293-mGPRC6A cells, whereas (B) L-ornithine increases cellular impedance only in tetracycline-induced cells. (C) Bovine OCN did not change cell impedance in either induced or un-induced FlpIn-TREx-HEK293-mGPRC6A cells. A similar lack of effect was shown for other OCN variants (*data not shown*).

To date, GPRC6A signal transduction studies have been largely limited to one of the above assay endpoints. To ensure that no hitherto unappreciated signalling pathway is activated downstream of murine GPRC6A with OCN, we evaluated the ligand pharmacology in a label-free assay of cellular impedance that represents a holistic measure of cellular activation. Using real-time monitoring of impedance (xCELLigence), we profiled the pharmacology of mGPRC6A expressed in FlpIn-TREx-HEK293 cells. Stimulation of endogenous P2Y purinergic receptors with ATP was used as a control for detecting activation of Gα_q_ protein-linked pathway(s). When either untreated or tetracycline-induced FlpIn-TREx-HEK293-mGPRC6A cells were stimulated with ATP, a concentration-dependent increase in cell index was observed, with a similar maximal impedance and potency in both non-expressing and mGPRC6A-expressing cells (Fig 4A). However, only mGPRC6A-expressing cells responded to L-ornithine (pEC_50_ = 4.2 ± 0.1; Fig 4B), with potency similar to that seen in inositol phosphate assays. Consistent with observations in other assays, none of the OCN variants tested modulated cell impedance in tetracycline-treated FlpIn-TREx-HEK293-mGPRC6A cells, suggesting that OCN peptides do not activate murine GPRC6A (Fig 4C and *data not shown*).

### GPRC6A function in entero-endocrine and pancreatic cells

**Fig 5:**
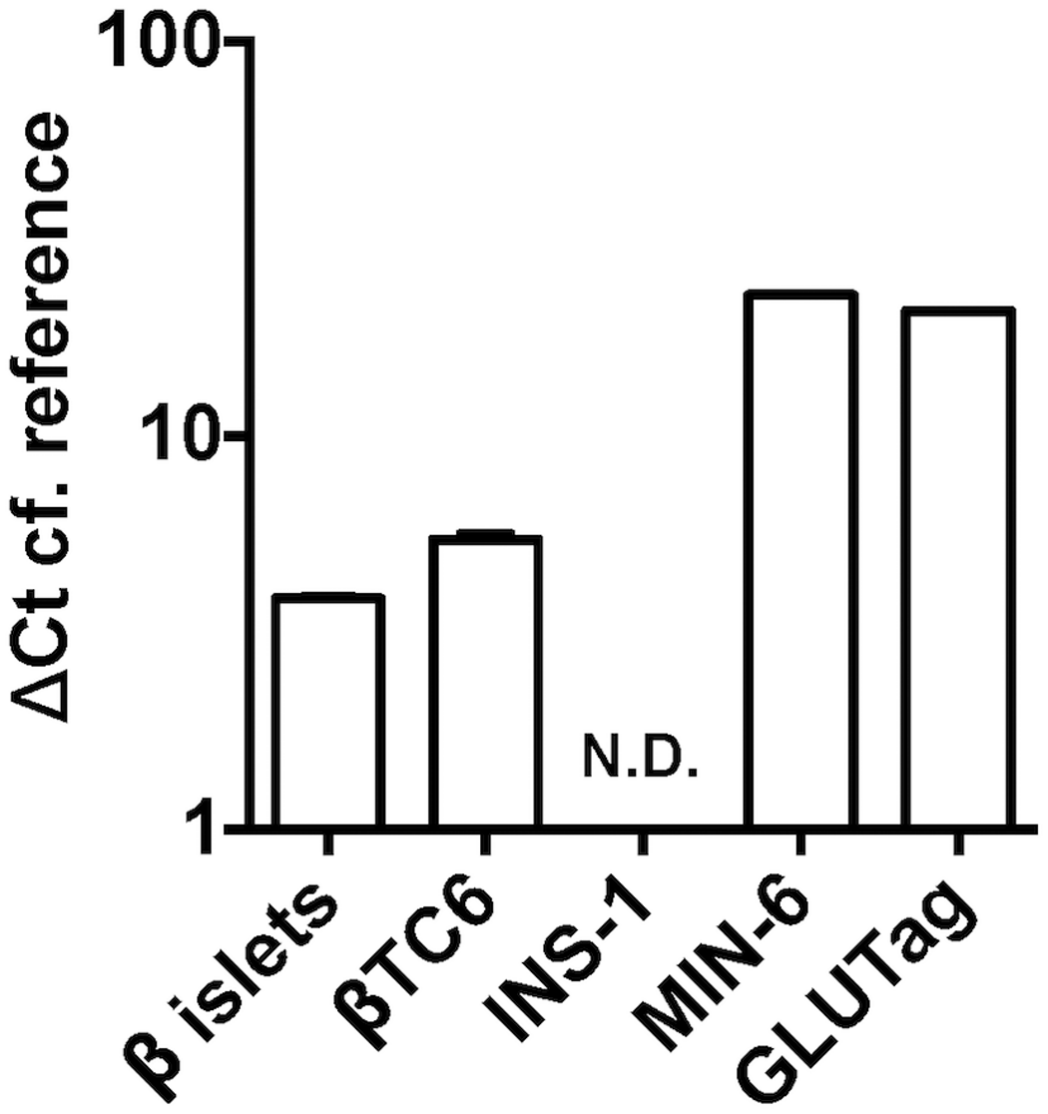
Determination of *Gprc6a* endogenous expression. *Gprc6a* mRNA expression in mouse primary islets, murine β-TC6, GLUTag, MIN6 and rat INS-1(832) cells as determined by qPCR or Taqman; N.D. = not detected.

Given the lack of response to OCN variants at recombinantly expressed mouse GPRC6A, we postulated that this might be due to the lack of appropriate cellular machinery or adaptor proteins in recombinant cells. Therefore we sought to test putative GPRC6A ligands in phenotypic assays in immortalised murine cells and mouse pancreatic islets that express GPRC6A. The GLUTag cell line was chosen as an entero-endocrine cell line as these cells have been previously demonstrated to respond to L-amino acids to secrete glucagon-like peptide (GLP)-1. MIN6 and β-TC6 cells were chosen as prototypical immortalised pancreatic β-cell lines for the study of insulin release. In parallel, we sought to evaluate insulin release in isolated mouse pancreatic islets. The three cell lines (as well as the islets) were shown to express *Gprc6a* mRNA to varying degrees (Fig 5). Finally we sought to identify a null cell line to demonstrate the specificity (or lack thereof) to any peptide effects; using qPCR we could not detect any *Gprc6a* mRNA expression in INS-1(832) cells, a rat insulinoma cell line.

**Fig 6:**
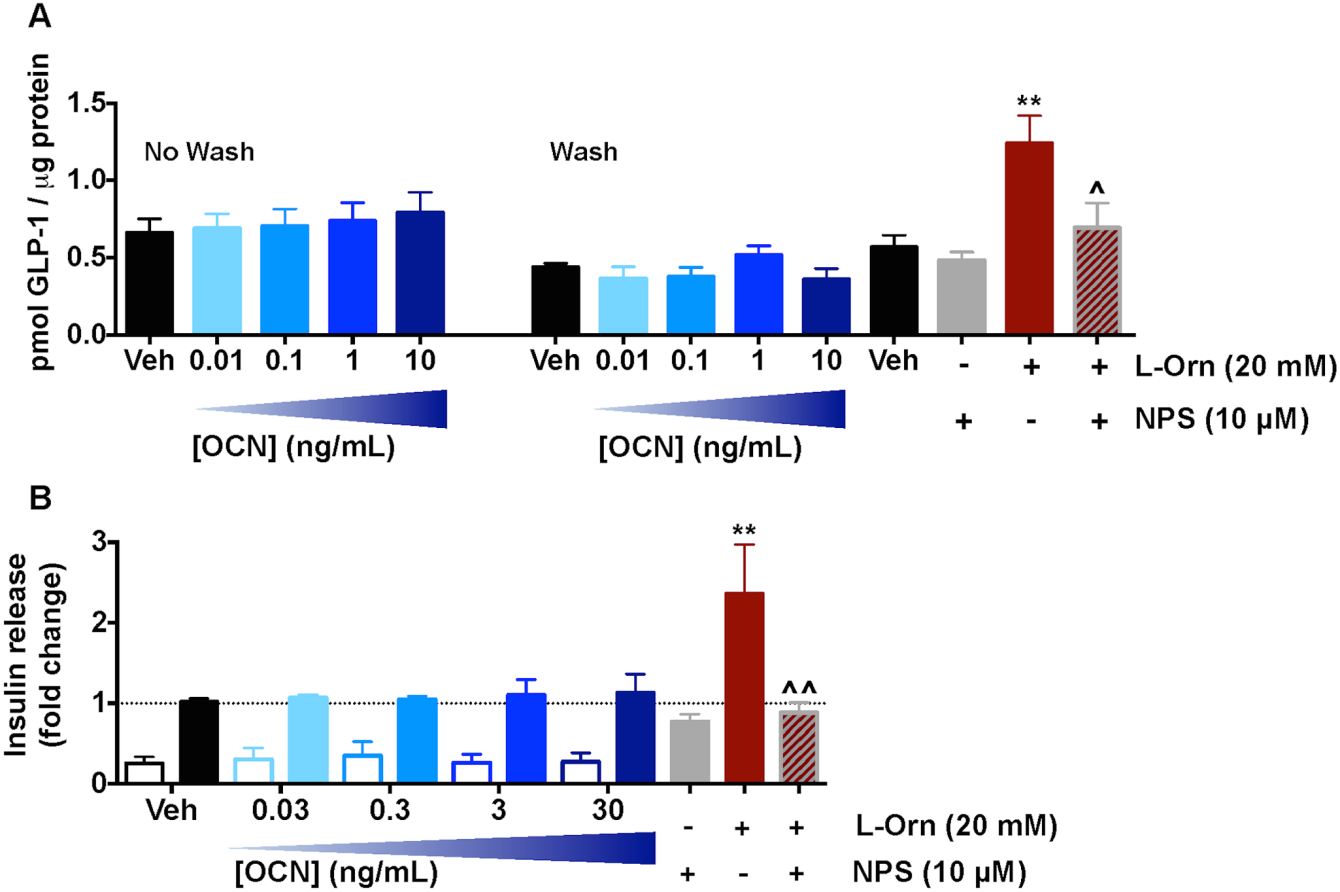
GPRC6A signalling in endogenously expressing cells. (A) L-ornithine (20 mM) significantly increases GLP-1 release from GLUTag cells (**P < 0.01 vs Vehicle, one-way ANOVA followed by Sidak’s multiple comparisons test). This effect is significantly reversed by NPS-2143 (^*P* < 0.05 vs. L-ornithine alone). Human synthetic OCN (0.01–10 ng/ml) did not significantly modulate GLP-1 release with or without cell washing. (B) High [glucose] significantly enhanced insulin secretion by MIN6 cells (*P* < 0.0001, two-way ANOVA). L-ornithine (20 mM), but not mouse synthetic OCN (acid form; 0.03–100 ng/mL), significantly increased glucose-sensitive insulin secretion (**P < 0.01 vs. Vehicle, one-way ANOVA for high [glucose] group followed by Sidak’s multiple comparison test. ^^ *P* < 0.01 vs. L-ornithine alone). Open bars, no glucose; filled bars, 16.7mM glucose. Data normalised to Insulin release in the presence of 16.7mM glucose.

In GLUTag cells, L-ornithine (20 mM) significantly increased GLP-1 release, an effect that was fully reversed by NPS-2143 (10 μM; Fig 6A). However, human synthetic OCN (acid form; 0.01–10 ng/ml) did not increase GLP-1 release whether the cells were washed (as for L-ornithine) or not (Fig 6A). A similar lack of effect was seen for the amidated form of the peptide (*data not shown*). In MIN6 cells, glucose significantly promoted insulin secretion (*P* < 0.0001, two-way ANOVA) and L-ornithine (20 mM) significantly increased glucose-sensitive insulin secretion (GSIS; *P* < 0.01 vs. Vehicle, one-way ANOVA for high [glucose] followed by Sidak’s multiple comparisons test; Fig 6B). This effect was fully reversed by NPS-2143 (10 μM; Fig 6B). In contrast, mouse synthetic OCN (0.03–30 ng/mL) did not affect insulin secretion at either glucose concentration (Fig 6B). A similar lack of effect was observed with recombinant mouse OCN (0.03–30 ng/mL; *data not shown*). To ensure that the lack of effect was not cell line-dependent, we also assessed OCN treatment on insulin release from β-TC6 cells and mouse pancreatic islets. Although both preparations released insulin in a glucose-sensitive manner (*P* < 0.001, two-way ANOVA for [glucose] effect in both studies), OCN (0.03–100 ng/ml) had no effect on insulin release at both glucose concentrations in either study (S3 Fig). The failure of OCN variants to stimulate GPRC6A-linked phenotypic endpoints in GLUTag, β-TC6 or MIN6 cells and mouse pancreatic islets casts further doubt as to whether GPRC6A is the direct target of this peptide.

**Fig 7:**
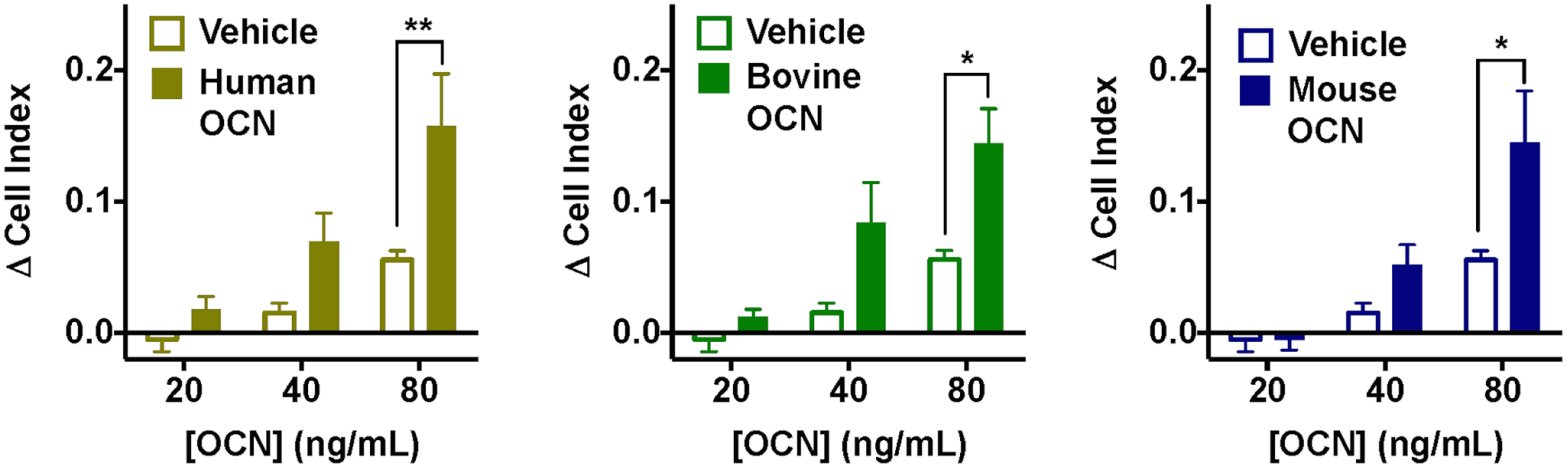
Measurement of cellular impedance in *Gprc6a*-negative rat INS-1(832) cells. Concentration-dependent increases in cell index (a measure of impedance) in INS-1(832) cells (that do not express *Gprc6a*) with synthetic human, purified bovine and synthetic mouse OCN. Statistical analysis performed by two-way ANOVA ([OCN] and treatment); there was a significant effect of both [OCN] and treatment for all studies; *P < 0.05, **P < 0.01 vs. Vehicle by Sidak’s multiple comparison test.

Finally we sought to examine the responsiveness of INS-1(832) cells, an immortalised rat pancreatic β-cell line that does not express GPRC6A mRNA (as determined by qPCR; Fig 5). For these studies we used the label-free xCELLigence assay to capture any cellular activity stimulated by ligands. Interestingly, in this cell line we were able to detect significant changes in cell impedance with purified bovine and synthetic mouse or human OCN (Fig 7). Thus, the only functional assay in which we were able to detect a response to OCN variants was in a cell line that does not appear to express GPRC6A.

## Discussion

In these studies we present a broad range of experimental evidence suggesting that murine GPRC6A is not a direct receptor target for the bone-derived peptide, osteocalcin (OCN). By examining cell signalling events downstream of recombinantly expressed mouse GPRC6A and phenotypic assays in cell lines endogenously expressing the *Gprc6a* transcript, we confirmed that GPRC6A is a receptor for basic L-amino acids, but were unable to detect any effect of OCN variants, irrespective of peptide species or source. GPRC6A has emerged in recent years as an interesting target due to its putative role in mediating the multiple *in vivo* effects of the under- or de-carboxylated form of the bone-derived peptide, OCN. A number of studies have demonstrated that OCN can increase pancreatic β-cell proliferation, insulin sensitivity and energy expenditure *in vivo*, as well as promoting insulin secretion *in vitro* and *ex vivo* [12, 20, 21]. Such effects may be beneficial in metabolic disorders such as type II diabetes, thus the direct target for the actions of OCN may represent an attractive point of therapeutic intervention. Furthermore, studies have shown that the beneficial effects of OCN are ameliorated or absent in *Gprc6a* KO mice, thus implicating this class C GPCR as the direct target receptor for OCN [12]. However, a definitive confirmation of this ligand-receptor pairing has proven somewhat elusive.

Whilst the ability of the receptor to be activated by basic L-amino acids is well accepted, several studies have reported that OCN can activate GPRC6A to cause intracellular calcium mobilisation [29], ERK1/2 phosphorylation [12], cAMP accumulation [8, 30] and increased β-cell proliferation [31] in GPRC6A-expressing cell lines or mouse pancreatic islets. The involvement of GPRC6A has been shown by transfection of receptor-specific siRNA, receptor blockade with a polyclonal antibody and, in the case of the islets, using tissue from KO mice.

A number of studies have specifically shown that OCN activates recombinantly expressed GPRC6A; two studies show that OCN (at approx. 40–80 ng/ml) promote ERK1/2 phosphorylation in HEK293 cells expressing GPRC6A (species not stated; [10, 12]). Interestingly, in one study this response was sensitive to inhibition of phospholipase C and protein kinase C, suggesting that it may be downstream of Gα_q_ activation. Other studies have showed that OCN stimulates cAMP accumulation in HEK293 cells transfected with GPRC6A at a concentration of either 3 ng/mL (species not stated for either receptor or peptide; [8]) or 60 ng/mL (mouse GPRC6A with human OCN; [11])

More recently, Brauner-Osborne and colleagues sought to comprehensively delineate the pharmacology of recombinantly expressed murine GPRC6A [13]. The major conclusions of this study were discrepant with the observations described above insomuch as GPRC6A was found to be primarily a Gα_q_ coupled, basic L-amino acid receptor. No effects on cAMP levels or ERK1/2 phosphorylation were observed and no agonist effect of OCN was evident in any of the assays profiled [13]. Given these discrepancies, we sought to evaluate the pharmacology of human and mouse GPRC6A in a range of cell signalling assays using L-amino acids and multiple variants of OCN, including recombinant, purified and synthetic peptides. The aim of the study was to provide a comprehensive evaluation of putative GPRC6A ligands in a range of assays, including G protein-dependent signalling assays in cells recombinantly expressing GPRC6A and metabolically-relevant phenotypic assays of GLP-1 release and insulin secretion in immortalised entero-endocrine and pancreatic β-cells, respectively.

Consistent with recent data identifying an intracellular retention motif present in the third intracellular loop of human GPRC6A [5], in our hands the human receptor expressed poorly at the cell surface of FlpIn-TREx-HEK293 cells compared to mouse GPRC6A. Consequently, our studies were limited to profiling this orthologue. Calcium mobilisation and inositol phosphate accumulation studies confirmed that murine GPRC6A was activated by basic L-amino acids, with L-ornithine, L-arginine and L-lysine the most potent agonists. However, robust inositol phosphate and calcium responses were only observed with transient co-expression of Gα_q_ or the promiscuously coupling Gα_qG66D_. There was no detectable modulation of cAMP accumulation or phosphorylation of ERK1/2 with L-ornithine, suggesting that mGPRC6A recombinantly expressed in FlpIn-TREx-HEK293 cells is primarily a Gα_q_ coupled receptor (Figs 3 and 4). These data are largely concordant with the findings of Jacobsen et al. (2013) [13], who drew a similar conclusion for the mouse GPRC6A recombinantly expressed in Chinese hamster ovary cells (although they did detect ERK1/2 phosphorylation in response to L-amino acids).

The major finding of our recombinant studies was the lack of effect of OCN variants to modulate any signalling pathway in FlpIn-TREx-HEK293 cells stably expressing mGPRC6A. Under- or de-carboxylated forms of OCN purportedly mediate activity at GPRC6A. Therefore, to obviate any issues of carboxylation status and/or species differences, we profiled multiple OCN variants in our *in vitro* assays (as described in Table 1) over a concentration range previously reported to show activity. Nevertheless, no agonistic or modulatory activity was detected at mGPRC6A, even in label-free assays of cell impedance that should capture any cellular signalling event (Fig 4B). Despite being performed in the same cell background (HEK293), these data are clearly discrepant with the cAMP and ERK1/2 phosphorylation assays previously reported for OCN at GPRC6A [8, 12], though the species of the receptor tested was not routinely defined in the previous studies. This lack of OCN function concurs with the findings of Jacobsen et al. (2013) [13], who detected only L-amino acid-mediated activation of mGPRC6A.

Given the range of OCN variants profiled, we propose that species and/or carboxylation status is unlikely to be a cause of the lack of activity in our assays. Other potential explanations are that the species of GPRC6A is critical or that HEK293 cells lack the necessary scaffolding and/or signalling proteins found in cells endogenously expressing GPRC6A. Unfortunately, human GPRC6A was not expressed at the cell surface in our cells, precluding a study of its pharmacology. However, we identified a range of immortalised murine entero-endocrine and pancreatic β-cell lines that express *Gprc6a* mRNA. De-carboxylated, but not carboxylated OCN has previously been shown to evoke GLP-1 release from the murine entero-endocrine STC-1 cell line [22] and GPRC6A has been implicated in L-amino acid-mediated GLP-1 release from GLUTag cells, an immortalised murine intestinal L-cell line [32]. Our studies confirmed the presence of *Gprc6a* transcript and demonstrated L-ornithine stimulated GLP-1 release from GLUTag cells in a NPS-2143-sensitive manner, indicative of a GPRC6A-mediated mechanism (Fig 6A). However, OCN treatment failed to increase GLP-1 release from this cell line. OCN has also been shown to increase insulin secretion from pancreatic islets [31] and engender ERK1/2 phosphorylation in βTC-6 cells [12], both in a GPRC6A-dependent manner. We confirmed the expression of *Gprc6a* transcript in the pancreatic β-cell lines MIN6 and βTC-6, as well as in mouse pancreatic islets (Fig 5). MIN6 cells displayed glucose-sensitive insulin secretion (GSIS), which could be increased by L-ornithine in an NPS-2143-senstive manner (Fig 6B). However, OCN treatment failed to induce GSIS, a profile that was replicated in both βTC-6 cells and isolated mouse pancreatic islets (S1 Fig). Thus we have been unable to replicate any of the reported GPRC6A-dependent *in vitro* activities of OCN.

Interestingly, the only cell line in which we could detect a cellular response to OCN variants was the rat insulinoma INS-1(832) cell line, in which bovine, mouse and human OCN displayed significant, concentration-dependent activity in the label-free cellular impedance assay as applied to the FlpIn-TREx-HEK293-mGPRC6A cells (Fig 7). However, no *Gprc6a* mRNA was detected in INS-1(832) cells by quantitative PCR. Whilst these data cannot prove that OCN is not a direct agonist of GPRC6A, it remains interesting that the only cell line in which our studies could detect effects of OCN is one in which there is no detectable transcript for the receptor.

In summary our studies have not been able to verify the purported agonist activity of OCN at mouse GPRC6A; *in vitro* cell signalling and phenotypic assays support the notion that murine GPRC6A is primarily a receptor for basic L-amino acids that signals *via* Gα_q_ proteins, in general agreement with the data of Jacobsen et al. (2013) [13]. The most parsimonious explanation for the data is that OCN does not directly activate mouse GPRC6A and that the observed *in vivo* effects of OCN reported in the literature may depend on GPRC6A, but are indirect and not due to an interaction with the receptor. This would account for many of the OCN effects being absent in *Gprc6a* KO mice. For the small number of reports of direct pharmacological effects of OCN on recombinantly expressed GPRC6A in HEK293 cells, as discussed above, only in one study is the species stated. It is possible that the human or rat receptor respond differently to OCN *in vitro* compared to the mouse clone that has been the focus of this and other studies [13]. Overall, our data do not support the notion of OCN as a direct agonist of murine GPRC6A, suggesting that the receptor may not represent a suitable therapeutic target to replicate the metabolically favourable effects of OCN *in vivo*.

## Supporting Information

S1 Fig Cell surface expression of mouse and human GPRC6A. FACS expression analysis with anti-c-*myc* (9E10) staining of FlpIn-TREx-HEK293 stably transfected with human or mouse c-myc GPRC6A using the pcDNA 5/FRT/TO or pIRES-puro6 expression constructs. Expression assessed as % of GPRC6A-expressing cells and total GPRC6A expression (fluorescence). Human GPRC6A did not express at the cell surface; the mouse orthologue was moderately expressed using the pcDNA 5/FRT/TO construct and at higher levels using the pIRES-puro6 vector.
(TIF)

S2 Fig Measurement of cAMP modulation in HEK293-pIRES-puro6-mGPRC6A cells. Neither L-ornithine nor OCN variants stimulate cAMP accumulation in HEK293 cells stably expressing pIRES-puro6-mGPRC6A (open circles); none of the ligands inhibit forksolin (3 μM)-stimulated cAMP accumulation in the same cell line (filled circles). OCN variants are as described in Table 1
(TIF)

S3 Fig Insulin secretion by β-TC6 cells or mouse pancreatic islets. (A) Glucose significantly enhanced insulin secretion by β-TC6 cells (*P* < 0.0001, two-way ANOVA), but there was no significant effect of either L-ornithine (20 mM) or human synthetic OCN (acid form; 0.03–100 ng/ml) to increase GSIS. (B) High [glucose] significantly enhanced insulin secretion by mouse pancreatic islets (*P* < 0.0001, two-way ANOVA). L-arginine (20 mM), but not human synthetic OCN (0.03–100 ng/ml) significantly increased GSIS (*P < 0.05 vs. Vehicle, two-way ANOVA followed by Sidak’s multiple comparisons test). Open bars, no glucose; filled bars, 16.7mM glucose.
(TIF)

S1 Table Summary of functional assays performed in cell lines recombinantly or endogenously expressing GPRC6A. (DOCX)
