# Supplementary figures and images for: Murine GPRC6A Mediates Cellular Responses to L-Amino Acids, but Not Osteocalcin Variants
Source: PLoS One. 2016 Jan 19;11(1):e0146846. doi: 10.1371/journal.pone.0146846 (PMC4718634; doi:10.1371/journal.pone.0146846)

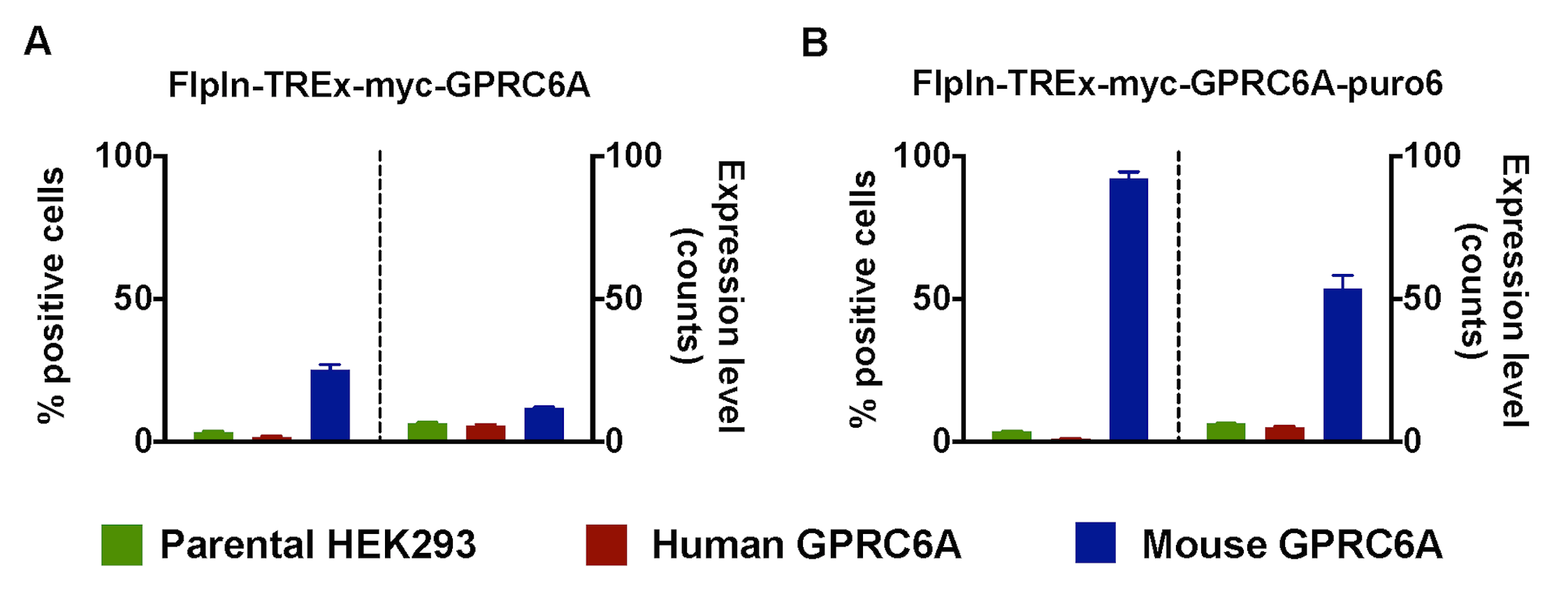

Supplement: S1 Fig — FACS expression analysis with anti-c-myc (9E10) staining of FlpIn-TREx-HEK293 stably transfected with human or mouse c-myc GPRC6A using the pcDNA 5/FRT/TO or pIRES-puro6 expression constructs. Expression assessed as % of GPRC6A-expressing cells and total GPRC6A expression (fluorescence). Human GPRC6A did not express at the cell surface; the mouse orthologue was moderately expressed using the pcDNA 5/FRT/TO construct and at higher levels using the pIRES-puro6 vector. (TIF) [file pone.0146846.s001.tif]

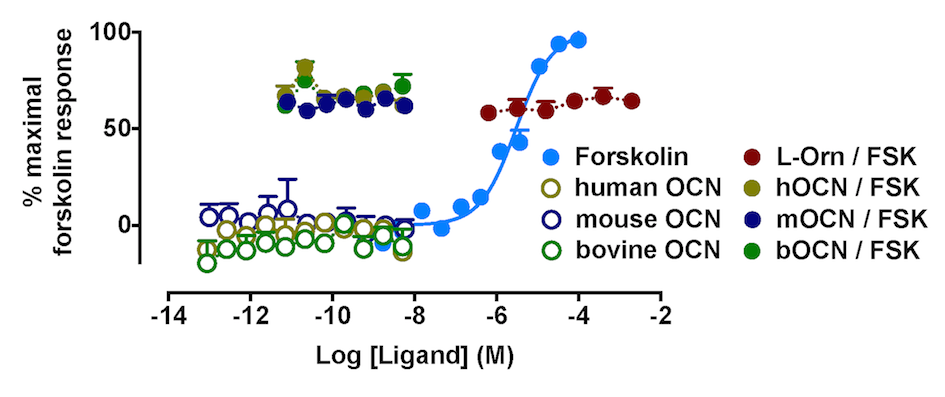

Supplement: S2 Fig — Neither L-ornithine nor OCN variants stimulate cAMP accumulation in HEK293 cells stably expressing pIRES-puro6-mGPRC6A (open circles); none of the ligands inhibit forksolin (3 μM)-stimulated cAMP accumulation in the same cell line (filled circles). OCN variants are as described in Table 1 (TIF) [file pone.0146846.s002.tif]

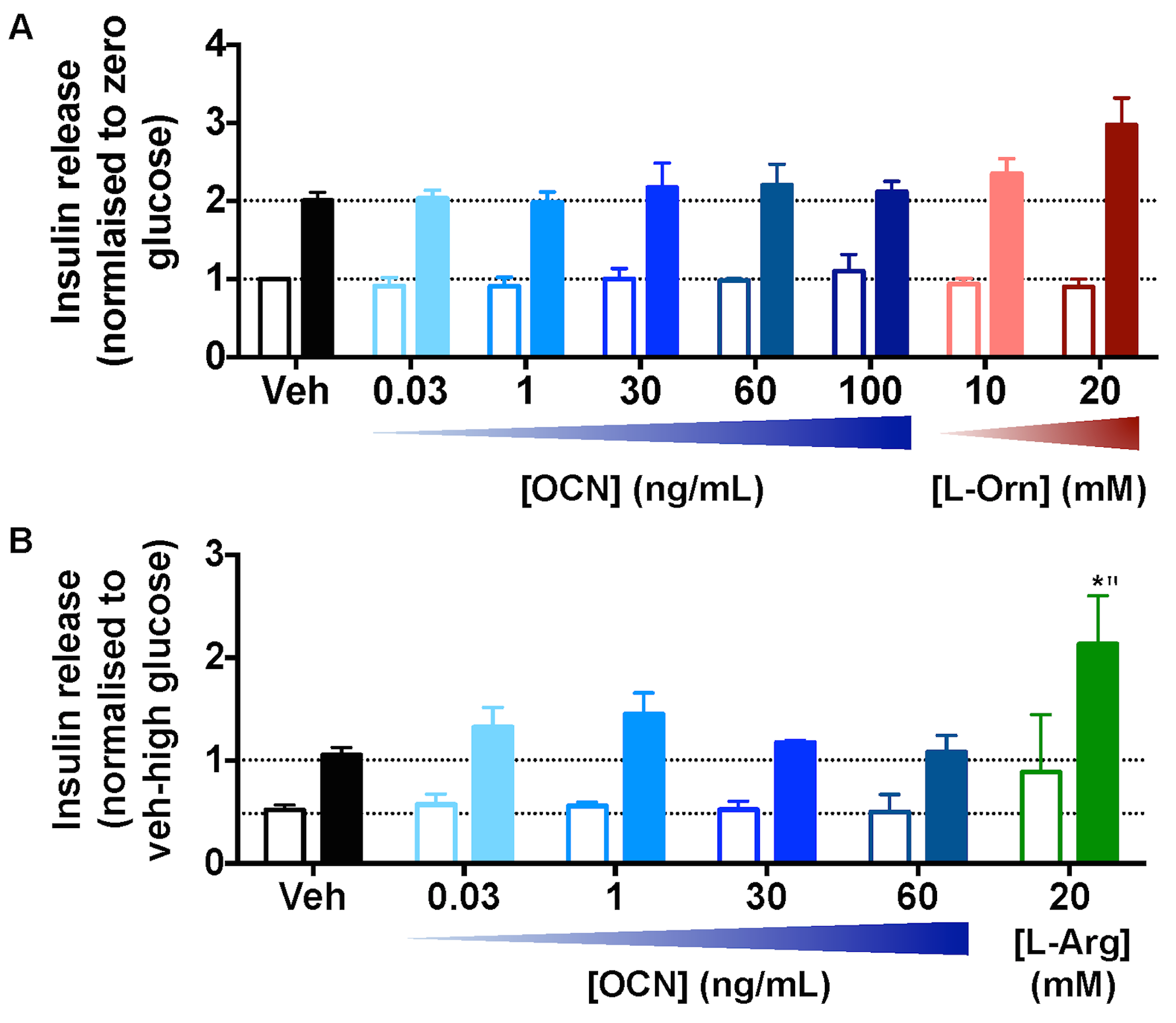

Supplement: S3 Fig — (A) Glucose significantly enhanced insulin secretion by β-TC6 cells (P < 0.0001, two-way ANOVA), but there was no significant effect of either L-ornithine (20 mM) or human synthetic OCN (acid form; 0.03–100 ng/ml) to increase GSIS. (B) High [glucose] significantly enhanced insulin secretion by mouse pancreatic islets (P < 0.0001, two-way ANOVA). L-arginine (20 mM), but not human synthetic OCN (0.03–100 ng/ml) significantly increased GSIS (*P < 0.05 vs. Vehicle, two-way ANOVA followed by Sidak’s multiple comparisons test). Open bars, no glucose; filled bars, 16.7mM glucose. (TIF) [file pone.0146846.s003.tif]
