# Supplementary material for: Murine GPRC6A Mediates Cellular Responses to L-Amino Acids, but Not Osteocalcin Variants
Source: PLoS One. 2016 Jan 19;11(1):e0146846. doi: 10.1371/journal.pone.0146846 (PMC4718634; doi:10.1371/journal.pone.0146846)
Supplement: S1 Table — (DOCX) [file pone.0146846.s004.docx]

| **Cell line** | **GPRC6A variant** | **Functional assay** | **Ref.** |
| --- | --- | --- | --- |
| tsA cells  (transient) | - hC6A/5.24 chimera - mGluR5 signal peptide - N-terminally tagged with c-myc | Ca^2+^_i_ - response to L-aa | [1] |
| *Xenopus laevis* oocytes (transient) | - hC6A/5.24 chimera or mC6A - mGluR5 signal peptide - N-terminally tagged with c-myc | Patch-clamp Ca^2+^_i_ activated Cl^-^ currents – response to L-aa |  |
| tsA cells (transient) | - hC6A/5.24 chimera, mC6A or rC6A - mGluR5 signal peptide - N-terminally tagged with c-myc - Co-transfected with Gα_q_G66D | [^3^H]IP turnover – response to L-aa | [2] |
| tsA cells (transient) | - mC6A - mGluR5 signal peptide - N-terminally tagged with c-myc - Co-transfected with Gα proteins | - [^3^H]IP turnover - co-transfection with Gα_qs5,_ Gα_qG66D,_ Gα_q or_ Gα_qG66Ds5_ enabled response to L-aa but Gα_qi_, Gα_qz_, Gα_qo_ did not - cAMP – no increase or decrease in response to L-Orn | [3] |
| *Xenopus laevis* oocytes  (transient) | - mC6A - mGluR5 signal peptide - N-terminally c-myc tagged | Patch-clamp Ca^2+^_i_ activated Cl^-^ currents – response to L-aa in the presence, but not in the absence of CaCl_2_ or MgCl_2_ |  |
| tsA cells (transient)  or  *Xenopus laevis* oocytes  (transient) | - mC6A untagged or N-terminally c-myc tagged (downstream of endogenous signal peptide) - 5.24/mC6A chimera | - Ca^2+^_i_ - response to L-aa for the 5.24/mC6A chimera but not mC6A - Patch-clamp Ca^2+^_i_ activated Cl^-^ currents – response to L-aa for c-myc-mC6a | [4] |
| HEK-293 cells  (transient) | - mC6A N-terminally c-myc tagged (downstream of endogenous signal peptide) | - cAMP accumulation in response to OCN | [5] |
| HEK-293 cells  (stable) | - GPRC6A | - pERK1/2 – response to Ca^2+^, Mg^2+^, Gd^3+^, Al^3+^, NPS R568 - SRE-reporter gene – response to CaCl_2_, SrCl_2_, MgCl_2_, GdCl_3_ and OCN | [6] |
|  | - hC6A - + Gα_qi5_ | Ca^2+^_i_ – response to Ca2+ Sr2+ |  |
| Murine MC3T3-E1  (endogenous) | - GPRC6A expression shown by RT-PCR | SRE-reporter gene – response to OCN |  |
| Murine MC3T3  (endogenous) | - GPRC6A expression shown by RT-PCR | pERK1/2 – response to Ca^2+^ | [7] |
| HEK293 cells  (transient) | - mC6A - mGluR5 signal peptide - N-terminally c-myc tagged - + Gα_qG66D_ | - [^3^H]IP or HTRF IP-One – response to L-Orn. Calindol and NPS 2143 non-competitive antagonists | [8] |
| HEK-293 cells  (stable) | - hC6A | - pERK1/2 – response to Ca^2+^, ZnCl_2_, OCN, Arginine, Testosterone-BSA | [9] |
| Human 22RV1 / PC-3  (endogenous) | - GPRC6A expression shown by RT-PCR | - pERK1/2 – response to Ca^2+^, ZnCl_2_, OCN, Arginine, Testosterone-BSA - Proliferation – response to Ca^2+^, OCN - PSA, Runx2 expression – response to OCN, Arg, R1881 - Chemotaxis (22RV1) – response to Ca^2+^, OCN |  |
| HEK-293 cells  (transient) | - mC6A | - pERK1/2 – response to Testosterone-BSA, R1881 - SRE-reporter gene – response to R1881, Ca^2+^ | [10] |
| Human 22RV1  (endogenous) | - GPRC6A expression shown by RT-PCR | - pERK1/2 – response to Testosterone-BSA, - Proliferation – response to Ca^2+^, OCN, Testosterone-BSA |  |
| HEK 293 cells  (transient) | - Unknown species GPRC6A | pERK1/2 - response to Ca^2+^, L-Arg, OCN. | [11] |
| Murine TC6  (endogenous) | - GPRC6A expression shown by RT-PCR | - pERK1/2, pPKD1 – response to OCN, L-Arg |  |
| Murine TM3 Leydig cells  (endogenous) | - GPRC6A expression shown by RT-PCR and IF | - pERK1/2 – negative - pCREB – response to OCN - Ca2+I negative - cAMP – response to OCN | [12] |
| CHO cells  (stable) | - mC6A - mGluR5 signal peptide - N-terminally c-myc tagged | - Ca^2+^_i_ negative - IP-One HTRF – response to L-Orn, Ca^2+^. No response to OCN, Testosterone. Compound 1 antagonist. - cAMP – no response to L-Orn, Ca^2+^, OCN or Testosterone - pERK – response to L-Orn. No specific response to Ca^2+^, OCN, Testosterone | [13] |
| Human tsA201 cells  (transient) | - mC6a - mGluR5 signal peptide - N-terminally c-myc tagged - + G_qG66D_ | - IP-One HTRF – response to L-Orn. No response to EC_25_ L-Orn + OCN |  |
| Human tsA201 cells  (transient) | - mC6a - mGluR5 signal peptide - N-terminally c-myc tagged - + G_qG66D_ | - [^3^H]-IP – response to L-Orn. Compound 1 and 3 antagonists. | [14] |
| Murine MA-10 Leyding cells  (endogenous) | - GPRC6A expression shown by WB and IF | - Release of 25-OH-Vit D or Testosterone – response to OCN - Ca^2+^_i_ – response to OCN - cAMP – no response to OCN - pERK1/2 – response to OCN | [15] |
| Murine 3T3-L1 adipocytes  (endogenous) | - GPRC6A expression shown by RT-PCR and WB | - cAMP – response to OCN - pERK1/2 – response to OCN - pCREB – response to OCN - Adiponectin, PPARγ expression – response to OCN | [16] |
| Murine GLUTag cells  (endogenous) | - GPRC6A expression shown by RT-PCR | - Ca^2+^_i_ – response to L-aa. Calindol antagonist - GLP-1 secretion – response to L-Orn. Calindol antagonist | [17] |
| Murine STC-1 cells  (endogenous) |  | - Ca^2+^_i_ – response to L-aa |  |
| HEK-293  (stable) | - mC6a | - cAMP – response to Testosterone, L-Arg - pERK1/2 – response to Testosterone | [18] |
| Human PC-3 cells  (endogenous) |  | - pERK1/2 – response to Testosterone, L-Arg |  |
| HEK-293  (transient) | - mC6a | - cAMP – response to L-Arg, OCN, Zn^2+^ | [19] |
| Human THP-1  (endogenous) | - GPRC6A expression shown by WB | - IL-1β secretion – response to Ca^2+^ - cAMP – no response to Ca^2+^ | [20] |
| FlpIn-TREx-HEK293 cells  (stable) | - mC6a - mGluR5 signal peptide - N-terminally c-myc tagged - + G_qG66D_ | - Ca^2+^_i_ – response to L-aa. No response to OCN - IP-One – response to L-aa. NPS2143 antagonist. No response to OCN - cAMP – no response to L-aa, OCN - pERK1/2 – no response to L-aa, OCN - Xcelligence – response to L-Orn. No response to OCN | Rueda et al. (2015) |
| Murine GLUTag cells  (endogenous) | - Expression shown by RT-PCR | - GLP-1 release – response to L-Orn. No response to OCN |  |
| Murine MIN6 or βTC6 cells  (endogenous) | - Expression shown by RT-PCR | - Insulin release – response to L-aa. No response to OCN |  |
| Rat INS-1 cells | - Expression not detected by RT-PCR or RNA seq | - xCELLigence – response to OCN |  |

1. Wellendorph P, Brauner-Osborne H. Molecular cloning, expression, and sequence analysis of GPRC6A, a novel family C G-protein-coupled receptor. Gene. 2004;335:37-46. doi: 10.1016/j.gene.2004.03.003. PubMed PMID: 15194188.

2. Wellendorph P, Burhenne N, Christiansen B, Walter B, Schmale H, Brauner-Osborne H. The rat GPRC6A: Cloning and characterization. Gene. 2007;396(2):257-67.

3. Christiansen B, Hansen KB, Wellendorph P, Brauner-Osborne H. Pharmacological characterization of mouse GPRC6A, an L-(alpha)-amino-acid receptor modulated by divalent cations. Br J Pharmacol. 2007;150(6):798-807.

4. Kuang D, Yao Y, Lam J, Tsushima RG, Hampson DR. Cloning and characterization of a Family C orphan G-protein coupled receptor. J Neurochem. 2005;93(2):383-91.

5. Oury F, Ferron M, Huizhen W, Confavreux C, Xu L, Lacombe J, et al. Osteocalcin regulates murine and human fertility through a pancreas-bone-testis axis. The Journal of clinical investigation. 2013;123(6):2421-33. doi: 10.1172/JCI65952. PubMed PMID: 23728177; PubMed Central PMCID: PMC3668813.

6. Pi M, Faber P, Ekema G, Jackson PD, Ting A, Wang N, et al. Identification of a novel extracellular cation-sensing G-protein-coupled receptor. J Biol Chem. 2005;280(48):40201-9.

7. Pi M, Zhang L, Lei SF, Huang MZ, Zhu W, Zhang J, et al. Impaired osteoblast function in GPRC6A null mice. Journal of bone and mineral research : the official journal of the American Society for Bone and Mineral Research. 2010;25(5):1092-102. doi: 10.1359/jbmr.091037. PubMed PMID: 19874200; PubMed Central PMCID: PMCPMC3153369.

8. Faure H, Gorojankina T, Rice N, Dauban P, Dodd RH, Brauner-Osborne H, et al. Molecular determinants of non-competitive antagonist binding to the mouse GPRC6A receptor. Cell calcium. 2009;46(5-6):323-32. doi: 10.1016/j.ceca.2009.09.004. PubMed PMID: 19836834.

9. Pi M, Quarles LD. GPRC6A regulates prostate cancer progression. The Prostate. 2012;72(4):399-409. doi: 10.1002/pros.21442. PubMed PMID: 21681779; PubMed Central PMCID: PMC3183291.

10. Pi M, Parrill AL, Quarles LD. GPRC6A mediates the non-genomic effects of steroids. The Journal of biological chemistry. 2010;285(51):39953-64. doi: 10.1074/jbc.M110.158063. PubMed PMID: 20947496; PubMed Central PMCID: PMC3000977.

11. Pi M, Wu Y, Quarles LD. GPRC6A mediates responses to osteocalcin in beta-cells in vitro and pancreas in vivo. Journal of bone and mineral research : the official journal of the American Society for Bone and Mineral Research. 2011;26(7):1680-3. doi: 10.1002/jbmr.390. PubMed PMID: 21425331.

12. Oury F, Sumara G, Sumara O, Ferron M, Chang H, Smith CE, et al. Endocrine regulation of male fertility by the skeleton. Cell. 2011;144(5):796-809. doi: 10.1016/j.cell.2011.02.004. PubMed PMID: 21333348; PubMed Central PMCID: PMC3052787.

13. Jacobsen SE, Norskov-Lauritsen L, Thomsen AR, Smajilovic S, Wellendorph P, Larsson NH, et al. Delineation of the GPRC6A receptor signaling pathways using a mammalian cell line stably expressing the receptor. The Journal of pharmacology and experimental therapeutics. 2013;347(2):298-309. doi: 10.1124/jpet.113.206276. PubMed PMID: 24008333.

14. Gloriam DE, Wellendorph P, Johansen LD, Thomsen AR, Phonekeo K, Pedersen DS, et al. Chemogenomic discovery of allosteric antagonists at the GPRC6A receptor. Chemistry & biology. 2011;18(11):1489-98. doi: 10.1016/j.chembiol.2011.09.012. PubMed PMID: 22118683.

15. De Toni L, De Filippis V, Tescari S, Ferigo M, Ferlin A, Scattolini V, et al. Uncarboxylated osteocalcin stimulates 25-hydroxy vitamin D production in Leydig cell line through a GPRC6a-dependent pathway. Endocrinology. 2014;155(11):4266-74. doi: 10.1210/en.2014-1283. PubMed PMID: 25093461.

16. Otani T, Mizokami A, Hayashi Y, Gao J, Mori Y, Nakamura S, et al. Signaling pathway for adiponectin expression in adipocytes by osteocalcin. Cellular signalling. 2015;27(3):532-44. doi: 10.1016/j.cellsig.2014.12.018. PubMed PMID: 25562427.

17. Oya M, Kitaguchi T, Pais R, Reimann F, Gribble F, Tsuboi T. The G protein-coupled receptor family C group 6 subtype A (GPRC6A) receptor is involved in amino acid-induced glucagon-like peptide-1 secretion from GLUTag cells. The Journal of biological chemistry. 2013;288(7):4513-21. doi: 10.1074/jbc.M112.402677. PubMed PMID: 23269670; PubMed Central PMCID: PMC3576058.

18. Pi M, Kapoor K, Wu Y, Ye R, Senogles SE, Nishimoto SK, et al. Structural and Functional Evidence for Testosterone Activation of GPRC6A in Peripheral Tissues. Mol Endocrinol. 2015:me20151161. doi: 10.1210/me.2015-1161. PubMed PMID: 26440882.

19. Pi M, Wu Y, Lenchik NI, Gerling I, Quarles LD. GPRC6A mediates the effects of L-arginine on insulin secretion in mouse pancreatic islets. Endocrinology. 2012;153(10):4608-15. doi: 10.1210/en.2012-1301. PubMed PMID: 22872579; PubMed Central PMCID: PMC3512028.

20. Rossol M, Pierer M, Raulien N, Quandt D, Meusch U, Rothe K, et al. Extracellular Ca2+ is a danger signal activating the NLRP3 inflammasome through G protein-coupled calcium sensing receptors. Nature communications. 2012;3:1329. doi: 10.1038/ncomms2339. PubMed PMID: 23271661; PubMed Central PMCID: PMC3535422.
